# Drift-Diffusion Analysis of Neutrophil Migration during Inflammation Resolution in a Zebrafish Model

**DOI:** 10.1155/2012/792163

**Published:** 2012-07-29

**Authors:** Geoffrey R. Holmes, Giles Dixon, Sean R. Anderson, Constantino Carlos Reyes-Aldasoro, Philip M. Elks, Stephen A. Billings, Moira K. B. Whyte, Visakan Kadirkamanathan, Stephen A. Renshaw

**Affiliations:** ^1^Department of Automatic Control and Systems Engineering, University of Sheffield, Sheffield S1 3JD, UK; ^2^MRC Centre for Developmental and Biomedical Genetics, University of Sheffield, Firth Court, Western Bank, Sheffield S10 2TN, UK; ^3^University of Sussex School of Engineering and Design Biomedical Engineering Research Group, Brighton BN1 9QT, UK; ^4^Department of Infection and Immunity, University of Sheffield, Sheffield S10 2JF, UK

## Abstract

Neutrophils must be removed from inflammatory sites for inflammation to resolve. Recent work in zebrafish has shown neutrophils can migrate away from inflammatory sites, as well as die in situ. The signals regulating the process of reverse migration are of considerable interest, but remain unknown. We wished to study the behaviour of neutrophils during reverse migration, to see whether they moved away from inflamed sites in a directed fashion in the same way as they are recruited or whether the inherent random component of their migration was enough to account for this behaviour. Using neutrophil-driven photoconvertible Kaede protein in transgenic zebrafish larvae, we were able to specifically label neutrophils at an inflammatory site generated by tailfin transection. The locations of these neutrophils over time were observed and fitted using regression methods with two separate models: pure-diffusion and drift-diffusion equations. While a model hypothesis test (the *F*-test) suggested that the datapoints could be fitted by the drift-diffusion model, implying a fugetaxis process, dynamic simulation of the models suggested that migration of neutrophils away from a wound is better described by a zero-drift, “diffusion” process. This has implications for understanding the mechanisms of reverse migration and, by extension, neutrophil retention at inflammatory sites.

## 1. Introduction

The fate of neutrophils following completion of the inflammatory programme is of critical importance for the outcome of episodes of acute inflammation and can determine whether there is prompt healing of a wound or the development of chronic inflammation and tissue injury. Neutrophils recruited to sites of inflammation may leave the site or die *in situ* [1]. The most widely accepted mechanism of neutrophil disposal is the programmed cell death or apoptosis, of the neutrophil followed by macrophage uptake and clearance (reviewed in [2]). Recently, other routes have been proposed; neutrophils may move away from the inflamed site into the bloodstream (“reverse transmigration” [3]), by migration through other tissues (“retrograde chemotaxis” or “reverse migration” [4–6]), or be lost into the inflammatory exudate [7, 8]. Current understanding of the process of reverse migration is reviewed elsewhere [9]. The uncertainty as to the *in vivo* fates of individual cells relates in part to the difficulty in following individual cells during inflammation resolution *in vivo*. The transgenic zebrafish model is emerging as a key model for the study of vertebrate immunity [10] and allows direct imaging and tracking of individual cells, and of populations of cells allowing their fate to be determined *in vivo*. Using a transgenic system, in which neutrophils express the fluorescent protein Kaede, notable for its ability to change fluorescence characteristics on exposure to light, we have assessed the fates of inflammatory neutrophils as inflammation resolves. Although others have used a similar system to label immune cell populations responding to much smaller stimuli [6], there has been no detailed study of the migratory patterns of neutrophils during inflammation resolution following tail transection.

Using dynamic modelling techniques based on the drift-diffusion equation, we tested the competing hypotheses that neutrophils were directed away from the wound region by proresolution agents produced locally or that they cease responding to existing chemokine gradients and redistribute as a feature of stochastic migratory behaviours.

## 2. Methods

### 2.1. Reagents, Zebrafish Lines and Maintenance

All reagents were from Sigma-Aldrich (Poole, UK) unless otherwise stated. Zebrafish were maintained according to standard protocols [11]. The *Tg(lyz: Gal4)i252* [12] and *Tg(UAS: Kaede)s1999t* [13] lines are described elsewhere.

### 2.2. Microscopy, Photoconversion, and Image Processing

For confocal microscopy, a Perkin Elmer Ultra *VIEW* VoX ERS 6FR Laser Confocal Imaging System (Perkin Elmer INC, USA) with an inverted Olympus IX81 microscope, equipped with six diode laser lines and a Yokogawa CSU-X1 spinning disk, was used to capture images on a 14-bit Hamamatsu C9100-50 Electron Multiplying-Charged Couple Device (EM-CCD) peltier-cooled camera (Hamamatsu Photonics Inc.), through an appropriate filter. For fluorescence microscopy, a Nikon Eclipse TE2000-U Inverted Compound Fluorescence Microscope (Nikon UK Ltd) was used with a Hamamatsu 1394 ORCA-ERA (Hamamatsu Photonics Inc.). Images were captured using Volocity build 5.3.2. A Perkin Elmer Ultra *VIEW* PhotoKinesis device, attached to the microscope described before, was used to photoconvert the Kaede protein using a 405 nm laser line. The device was calibrated using a glass microscope slide (Menzel-Gläzer) covered with fluorescent highlighter ink (Stabilo Boss) as a photobleachable substrate (according to manufacturers instructions). Photoconversion was performed using 40% laser energy for 120 cycles of the 405 nm laser line. The embryos were then released from the agarose gel and transferred to fresh E3. The petri dishes containing the embryos were wrapped in tinfoil to prevent background photoconversion. At the timepoints indicated, embryos were again mounted and widefield fluorescence Z-stacks taken. Neutrophil segmentation was performed in Volocity based on fluorescence intensity, size, and “separate touching objects” feature. The XY position of each fluorescent cell at each timepoint was determined.

### 2.3. Dynamic Modelling of Neutrophil Behaviour

Neutrophil centroid coordinates in time were exported into Matlab (MathWorks, MA), for analysis. To describe quantitatively the population dynamics of neutrophils, drift-diffusion and pure-diffusion variants of the simple random walk model were used ([14] see Supplementary Material for full details available online at doi: 10.1155/2012/792163). Using parameters identified in these models, the behavior of each model was tested by simulation using a Monte Carlo procedure and the distribution of simulated cell populations compared to the observed data.

## 3. Results and Discussion

### 3.1. Characterising the Process of Reverse Migration *In Vivo*

**Figure 1:**
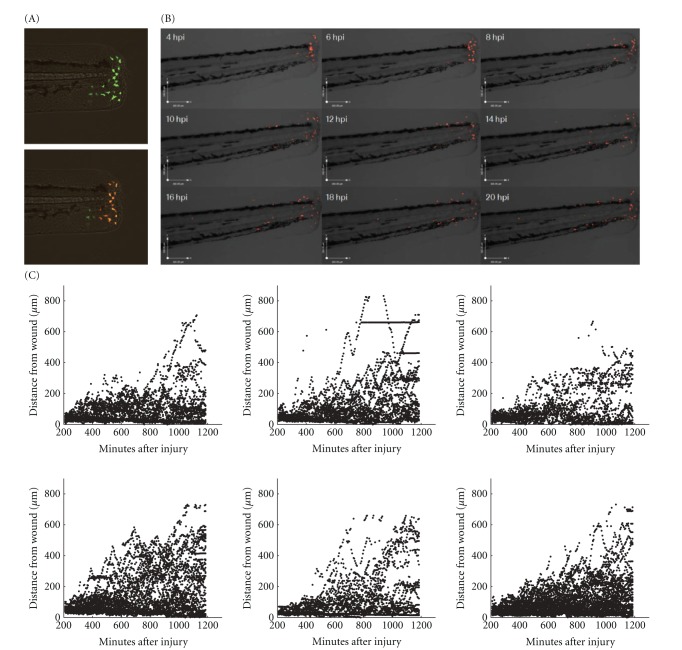
Inflammatory neutrophils exhibit restricted migration away from the site of tissue injury. 3 dpf embryos from transgenic zebrafish expressing Kaede in neutrophils were subjected to tailfin transection under anaesthesia using a sterile scalpel. The embryos were recovered for 4 hours. At four hours after injury the embryo was mounted in 0.5% low melting point agarose for imaging on a Laser Confocal System (Perkin Elmer Inc). The PhotoKinesis device was then used to photoconvert all neutrophils present within the tip of the tailfin. Photoconversion was carried out according to the methods described (120 cycles of 40% 405 nm laser energy), and time-lapse videomicroscopy was performed using a TE2000 fluorescent inverted microscope (Nikon). (a) Composite images of DIC overlaid with the red and green fluorescence channels showing a representative zebrafish tail before (above) and after (below) photoconversion. (b) A montage of DIC images overlaid with the red fluorescence channel at then timepoints indicated after tailfin injury. The redistribution of photoconverted cells can be clearly seen over time. (c) For each neutrophil in six individual fish, the distance from the wound was calculated using algorithms within Volocity and plotted against time.

Reverse migration, either into the circulation or back into tissues, has been described in the zebrafish model [4–6, 15]. In order to define the fates of inflammatory neutrophils, we photoconverted neutrophils in the immediate vicinity of the wound edge (approximately 80 microns) (Figure 1(a)) at defined periods after initiation of inflammation by tailfin transection. Time-lapse videomicroscopy was then performed on a compound fluorescent microscope, and the position of individual cells tracked in Volocity. Kaede protein and its photoconverted form remained stable and detectable well beyond the duration of these experiments (data not shown).

In over 500 hours of observation, no photoconverted neutrophil was ever seen to have left the fish from the wound, to have entered the circulation, or to have migrated via the circulation into a distant site. Neutrophils were seen to migrate away from the site of injury from around 8 hours after injury (Figure 1(b)). Photoconverted neutrophils can be seen to migrate away from the site of injury over the 16-hour time-lapse (Supplementary Movie 1 available online at doi:10.1155/2012/792163). At 4 hpi, neutrophils are densely accumulated around the site of injury, but over the duration of the time-lapse a population of neutrophils appears to spread into the surrounding tissue. Plots of the distance of each cell from the wound edge against time reveal a distinct pattern of neutrophil movement: neutrophils appear to be constrained in their behaviour, gradually increasing their mean distance from the wound, at a rate slower than their maximum speed would permit (Figure 1(c)). The differences between these findings and those of other groups [6] have many potential explanations, including the use of different promoters, different wounding protocols, and different labelling systems.

### 3.2. Neutrophils Continue to Be Recruited after Peak Inflammation

**Figure 2:**
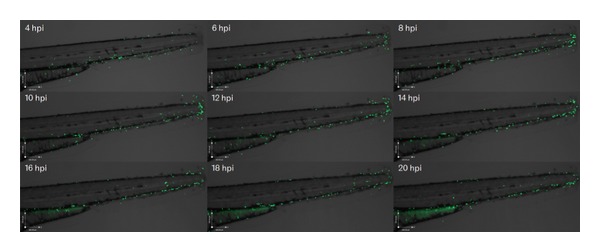
At peak inflammation, new neutrophils are recruited to the site of injury. Photomontage generated from the time-lapse data used in Figure 1(b), and Supplemental Movie 1, imaged using the GFP filterset, showing neutrophil recruitment to the site of injury over the same timespan. Green neutrophils can be seen to accumulate at the site of injury between 6 and 14 hours after injury.

In mammalian inflammation, neutrophil influx ceases early in the inflammatory response, at least in rabbit models of pneumonia [16]. The neutrophil Kaede model allows us to distinguish the behaviour of neutrophils present at the site of inflammation from the behaviour of those cells in the process of being recruited. The montage in Figure 1 shows only the red photoconverted neutrophils. During the time-lapse, images were also taken using filter sets optimised for green fluorescence. The green neutrophils identified were cells that were not present at the site of injury at 4 hpi. The behaviour of these cells shows that neutrophils are still recruited to the site of inflammation at four hours after injury (Figure 2). There are no green neutrophils seen at the site of injury at 4 hpi because all the cells present have been photoconverted. There is an accumulation of green neutrophils at the site of injury from 6 hpi until 14 hpi. Following this, the number of green neutrophils at the site of injury falls. Where individual cells can be seen and followed over time, the pattern of accumulation of neutrophils during inflammation can be accurately determined. This technique has increased sensitivity for detecting continued influx compared to mammalian labelled-cell techniques, and this may explain the differences seen from rabbit pneumonia models where influx is no longer detectable shortly after initiation of the inflammatory episode [17].

### 3.3. Neutrophils Actively Migrate (“Drift”) toward a Wound

**Figure 3:**
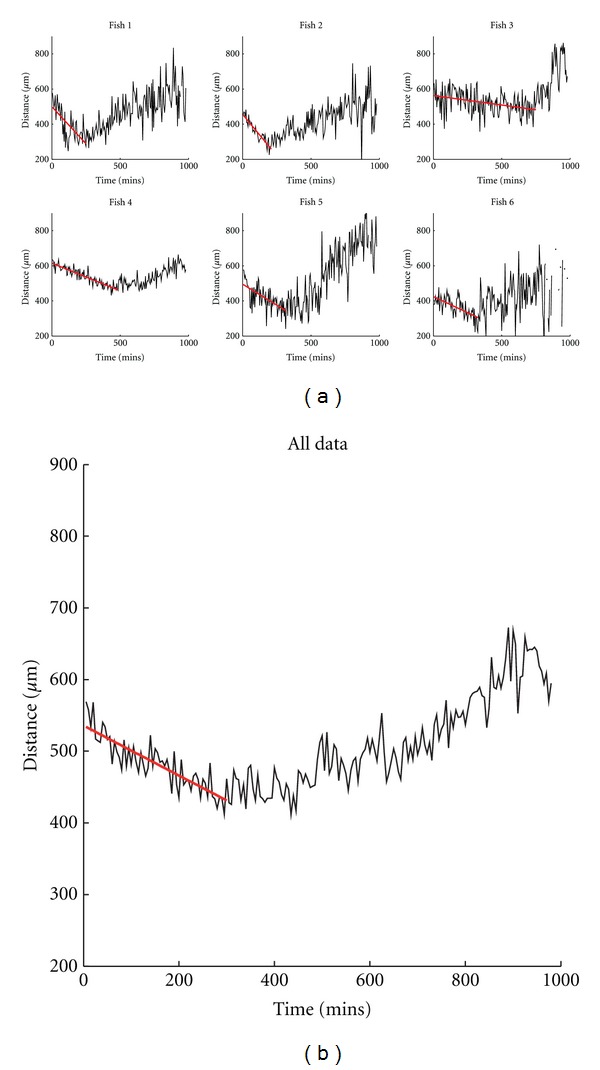
Nonphotoconverted neutrophils actively migrate into the wound region. (a) Variation over time of mean cell distance from the wound for the nonphotoconverted (green) neutrophils, observed in each subject 1–6 (black line). Overlaid on each graph is the prediction of mean distance obtained from the linear model used to characterise the initial drift (red line). The time is measured from the start of observations which commenced 4 hours after injury. The cell count in subject 6 (bottom right) was low and sometimes zero near the end of the dataset, which explains the missing sections. (b) Data and model combined over all subjects.

Random walk models are often used in biology to describe the movement dynamics of individuals and populations [14, 18] and particularly for cell movement patterns [19–21]. Over short timescales neutrophils exhibit correlated random walk behaviour. However, these local correlations decay over time. The time between our data observations is greater than typical neutrophil persistence times [22] and thus we are able to ignore these local correlations and apply a simple random walk model [18]. To identify any global directional bias apparent in the movement of neutrophils, the simple random walk model was applied to aggregate data. The contribution of active recruitment (chemotaxis) of neutrophils and its reverse (fugetaxis) were examined by establishing the positions of all neutrophils at 4 hours following tail fin transection and modelling their behaviour using a drift-diffusion equation. Non-photoconverted neutrophils were examined to determine the behaviour of neutrophils not at the wound site at the time of photoconversion. Fitting the drift-diffusion equation to the dataset treats the neutrophils as point objects and asks whether they are behaving like simple particles redistributing stochastically (“diffusion”) or whether there is an element of active movement towards or away from a chemical gradient (chemotaxis or fugetaxis). The equation (full description in supplemental data) generates a value for the drift co-efficient, for which non-zero values reflect an active rather than purely random migration. The drift was estimated from the linear relationship between time and mean cell distance from the wound (Figure 3). For 6 independent experiments, the coefficient estimates ranged in value from 0.11 to 0.95 *μ*m/min (Table 1). As expected, in all cases cell populations demonstrated active drift toward the wound, consistent with migration directed by a chemotactic process.

### 3.4. Migration of Neutrophils away from a Wound Is Better Described by a Zero-Drift, “Diffusion” Process

**Figure 4:**
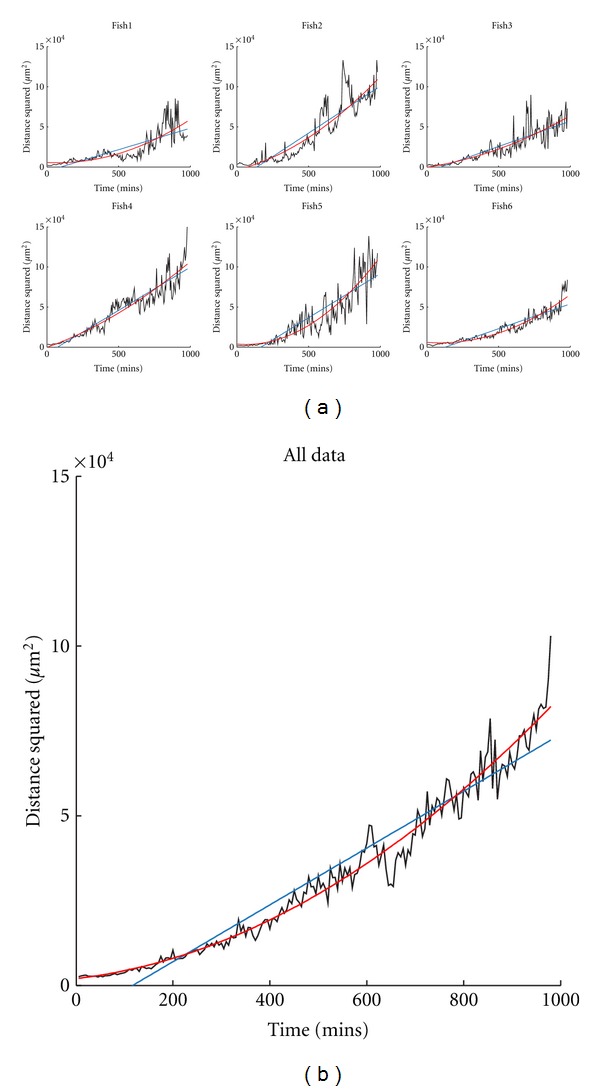
Inflammatory neutrophil behaviour can be fitted by pure-diffusion and drift-diffusion models. (a) Plots of mean squared cell distance from the wound against time for the photoconverted (red) neutrophils for datasets 1–6. Also shown on each plot are the fits for the linear model corresponding to pure-diffusion with zero drift (blue line) and for the drift-diffusion model (red line). (b) Data and models combined over all subjects.

**Figure 5:**
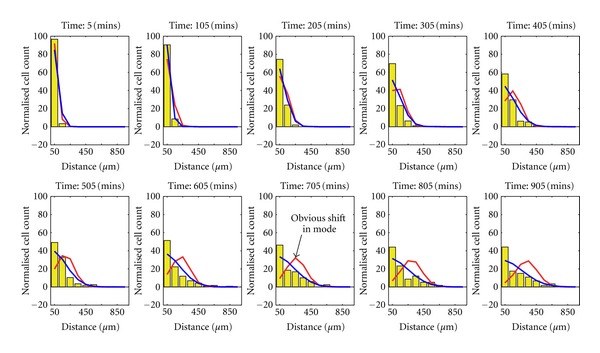
Simulation reveals a pure-diffusion model to be a better fit to the real data. Both the drift-diffusion model (red line) corresponding to drift (0.26 *μ*m/min) and diffusion (8.0 *μ*m/min) and the pure-diffusion model (blue line) corresponding to diffusion (41.8 *μ*m/min) were simulated 1000 times. The simulations were used to produce a distribution for the spatially binned data of each model. The mean values of cell distribution over space are shown by the red and blue lines, respectively (in terms of distance from the wound). Overlaid on these is a corresponding histogram representation (yellow) of the real data (combined over all fish). The histogram bins have width 100 *μ*m and are centered at 50 *μ*m to 950 *μ*m from the wound. The pure-diffusion model shows a correct qualitative prediction of cell distribution whereas the drift-diffusion model predicts that the population mode moves away from the wound over time, in contrast to the observed data.

The same analysis was performed for photoconverted cells present at the site of the wound at the time of photoconversion, 4 hours following the tailfin transection (Table 2). Drift-diffusion and pure-diffusion model fits are compared in Figure 4. Mathematical testing of the fit of the two models suggested that the drift-diffusion model fitted better with the data, but we were alert to the possibility that drift-diffusion models might appear superior due to the better ability of quadratic fits to model real, noisy data than simple linear fits. Using modeled data comparing the predicted distributions of neutrophils over time by applying drift-diffusion versus pure diffusion models gave a dramatic result: the cell population mode of the drift-diffusion model moved away from the wound over time (Figure 5, red line), in contrast to the observed data, where the mode remained close to the wound (Figure 5, yellow bars). The pure-diffusion model accurately captured this qualitative behavior, more accurately reflecting the observed distribution of neutrophils over time (Figure 5, blue line), suggesting that stochastic redistribution might best describe the pattern of neutrophil behavior during inflammation resolution.

For the larger wounds used in these studies, our data support a stochastic redistribution of neutrophils during inflammation resolution. However, to definitively prove this will require more advanced modelling techniques. For smaller wounds, different principles may apply. Previous studies have suggested that neutrophils leaving the wound follow the same dynamics as those arriving, having the same velocity and directionality [15]. However, those data rely on preselection of tracks directly leaving the wound, and may give different results to studies considering the whole population of cells.

This approach uses static point data for each neutrophil; an alternative approach would be toinvestigate the dynamics using individual track data.Such an approach has been applied to proteins in living cells [23, 24] and to *in vivo* melanoma cell tracks [25]. Care is needed when considering cell tracks as a naive approach could misrepresentshort-term correlations in track direction as biased migration. In addition, to identify tracks requires faster sampling of observations which must be balanced against total experiment runtime. 

Although the pure-diffusion model appears to fit the data well, it consistently underestimates the number of photoconverted cells remaining adjacent to the wound, suggesting some cells are actively retained at the wound site. To completely address this will require the development of systems incorporating multiple models to reflect the dynamic mix of neutrophil behaviours present within a single population. 

## 4. Conclusions

From this analysis, we conclude that the two key neutrophil migratory behaviours regulating neutrophil numbers during the inflammatory response—movement of neutrophils in and out of wounds—are qualitatively different processes. Neutrophils are recruited actively towards the site of injury (“drift”), but as inflammation resolves, their movement away is better modelled by stochastic redistribution (“diffusion”). This has implications for our understanding of how neutrophils might be retained at sites of inflammation in disease states.

## Figures and Tables

**Table 1:** Estimated drift coefficients for the model of drift-diffusion describing cell migration toward the wound.

Dataset	Drift coefficient (std dev.)
(1)	−0.85 (0.13)
(2)	−0.95 (0.06)
(3)	−0.11 (0.02)
(4)	−0.32 (0.02)
(5)	−0.48 (0.08)
(6)	−0.37 (0.06)
All data	−0.35 (0.03)

**Table 2:** Estimated coefficients for the drift-diffusion model and pure-diffusion model of cell migration away from the wound (standard deviation is given in brackets). An *F*-test value >5 indicates that the drift-diffusion model should be preferred to the pure-diffusion model.

Dataset	Drift-diffusion model		Pure-diffusion model	* F*-test
	Drift coefficient	Diffusion coefficient	Diffusion coefficient
(1)	0.25 (0.05)	−4 (10)	27 (2)	38
(2)	0.27 (0.07)	23 (15)	56 (4)	28
(3)	0.19 (0.05)	13 (10)	32 (3)	14
(4)	0.21 (0.05)	32 (11)	54 (3)	14
(5)	0.35 (0.07)	−8(14)	55 (4)	82
(6)	0.27 (0.03)	−7 (6)	31 (2)	145
All data	0.26 (0.02)	8 (3)	41.8 (0.10)	267
